# Home-Based Intervention Tool for Cardiac Telerehabilitation: Protocol for a Controlled Trial

**DOI:** 10.2196/47951

**Published:** 2025-01-22

**Authors:** Francesca Mastorci, Maria Francesca Lodovica Lazzeri, Lamia Ait-Ali, Paolo Marcheschi, Paola Quadrelli, Massimiliano Mariani, Rafik Margaryan, Wanda Pennè, Marco Savino, Giuseppe Prencipe, Alina Sirbu, Paolo Ferragina, Corrado Priami, Alessandro Tommasi, Cesare Zavattari, Pierluigi Festa, Stefano Dalmiani, Alessandro Pingitore

**Affiliations:** 1 Clinical Physiology Institute Consiglio Nazionale delle Ricerche Pisa Italy; 2 Fondazione Toscana G Monasterio Pisa Italy; 3 GPI SpA Pisa Italy; 4 Computer Science Department University of Pisa Pisa Italy

**Keywords:** cardiac rehabilitation, exercise, patient education, patient-centered approach, eHealth, artificial intelligence

## Abstract

**Background:**

Among cardiovascular diseases, adult patients with congenital heart disease represent a population that has been continuously increasing, which is mainly due to improvement of the pathophysiological framing, including the development of surgical and reanimation techniques. However, approximately 20% of these patients will require surgery in adulthood and 40% of these cases will necessitate reintervention for residual defects or sequelae of childhood surgery. In this field, cardiac rehabilitation (CR) in the postsurgical phase has an important impact on the patient by improving psychophysical and clinical recovery in reducing fatigue and dyspnea to ultimately increase survival. In this context, compliance with the rehabilitation program is a key element for the therapeutic benefits of the program. The increase of mobile health care devices and software has greatly extended self-care capabilities across the spectrum of health care activities. Moreover, the possibility of telemonitoring the progress of this self-care provides elements of empowerment and awareness of one’s state of health. As a branch of telehealth, CR can be optimized and facilitated using remote telemedicine devices.

**Objective:**

The principal goal of the Innovation in Postoperative Rehabilitation Training and Monitoring (IPOTERI) study is to design, realize, and test a composite and integrated system for postsurgical rehabilitation therapies at home specialized for cardiac surgery. The secondary aims are to implement the system in a “real-life” context of postcardiac surgical rehabilitation, and to create a data set and a data collection methodology to prototype data analytics algorithms and artificial intelligence techniques for customizing the rehabilitation pathway.

**Methods:**

The IPOTERI method consists of a telemonitoring platform that guarantees continuity of postoperative care, an intelligent home station based on an Android app for the patient with a user-friendly interface to record vital signals (electrocardiogram, blood pressure, oxygen saturation, and body weight) and access the planning of rehabilitation activities, and a decision support system that communicates with hospital medical records to transmit alerts and specific support information for the formulation and updating of the treatment and care plan.

**Results:**

The pilot test started in June 2023 (protocol number 20406/2021) including 50 patients who will be monitored for 12-14 weeks using the developed platform, as described in the Procedures subsection of the Methods section.

**Conclusions:**

The IPOTERI approach, based on the processing of data recorded during the monitoring of telemedicine devices used at home during the postsurgical rehabilitation of a cardiac patient, together with clinical data from the perioperative and postoperative periods could have positive effects on adherence to the rehabilitation program and clinical improvement as well as result in overall improvement of quality of life.

**International Registered Report Identifier (IRRID):**

DERR1-10.2196/47951

## Introduction

### General Background

Cardiovascular diseases (CVDs) represent the leading cause of death worldwide, which are responsible for 17 million deaths annually according to the World Health Organization [1]. This figure is expected to increase, as it is estimated that 23 million people will die from CVDs worldwide in 2030, accounting for 31% of all deaths globally [2]. People with CVDs typically require steady assistance and management by both the patient and their caregivers, mainly due to comorbidity with other pathologies, including high blood pressure, diabetes, and hyperlipidemia.

Among CVDs, adult patients with congenital heart disease represent a population that has been continually increasing, mainly owing to the improvement of the pathophysiological framing, including the development of surgical and reanimation techniques that have contributed to enhanced survival of these patients into adulthood [3]. Recent studies indicate that approximately 20% of patients with congenital heart diseases will require surgery in adulthood and 40% of these cases will necessitate reintervention for residual defects or sequelae of childhood surgery [4]. In these patients, the benefits of cardiac rehabilitation (CR) techniques are particularly important and clinically impactful during the postoperative period.

### Clinical Background of the CR Field

CR is an evidence-based program that is considered part of secondary prevention, which is focused on three main aspects: patient education, health behavior modification, and exercise training [5]. CR programs primarily target patients with ischemic heart disease, heart failure, or following cardiac surgery to reduce mortality and morbidity. A complete rehabilitation plan should include both a physical and psychological agenda, encompassing clinical evaluation, pharmacotherapy, psychological support, training, assessment and reduction of risk factors (both physical and psychological factors), and patient education for lifestyle modification. Thus, the gold standard of CR is an approach integrating health behaviors such as increased physical activity, improved dietary habits, and smoking cessation, along with promotion of medical adherence and psychosocial well-being strategies. There are multiple reported benefits of CR in patients following cardiac surgery. Several studies have documented that in addition to speeding up psychophysical recovery, CR improves clinical parameters, namely by reducing fatigue and dyspnea, ultimately resulting in increased survival with a drop in mortality at 10 years after surgery reaching up to 14% [6]. Interestingly, the benefit in terms of major event-free survival correlates with the number of rehabilitation training sessions to improve quality of life and reduce cardiovascular risk factors [7-9].

Although the clinical importance of CR is officially recognized, the proportion of patients admitted to a rehabilitation program remains small and there is overall low patient compliance [10]. Several factors may contribute to this low enrollment rate in CR programs, including physicians’ and patients’ approaches toward CR. In general, the percentage of patients who adhere to CR is variable, depending mainly on age, gender, and the presence of preexisting neurological complications [11]. Moreover, in addition to poor adherence, the capacity of services for CR in primary care is hindered due to limitations of human resources and adequate space in hospitals to support rehabilitative programs.

To overcome these problems, an alternative method to the traditional center-based CR (CBCR) model is home-based CR (HBCR), which is a new method that can be carried out in different settings, ranging from the home to a park, owing to the support of telemedicine.

Evidence from published studies comparing CBCR and HBCR has revealed that in some cases, the results are overlapping with improvements in 3- to 12-month clinical outcomes and no differences in hospitalization rates [12]. In particular, benefits have been documented regarding functional improvement, managing risk factors, and well-being perception. With respect to compliance, HBCR is associated with a lower dropout rate and higher degree of responsiveness and perseverance compared to CBCR [13]. For these reasons, an important aim will be to extend the applicability of HBCR to different patient subgroups, including older adults, women (who tend to have relatively poor compliance [14]), and high-risk patients. Based on current evidence, it is reasonable to believe that HBCR can be targeted to patients in a stable state with low to moderate risk. Furthermore, despite international guidelines recommending CR, there are no unique programs tailored according to pathology or country of origin. For example, in Italy, CR is part of the rehabilitation plan in the hospital; however, according to the Italian Survey on Cardiac Rehabilitation (ISYDE), the Italian cardiology rehabilitation network is only able to offer the intervention to approximately 60,000 patients per year against an estimated demand of more than 300,000 patients per year [15]. To reduce the imbalance between supply and demand, the ISYDE project was developed with the goal of obtaining a detailed snapshot of the number, distribution, facilities, staffing levels, organization, and program details of CR units in Italy.

Considering the increasing burden of CVD, from an economic perspective and taking into account the increasing pressures facing health systems, cost-effectiveness is an essential consideration for CR program development. Previous data on economic evaluations of HBCR have found positive effects compared to traditional care, although the heterogeneity in methodologies may limit the validity of these findings [16,17].

### Mobile Health for CR

Advances in mobile networks and the proliferation of smartphones and tablet devices constitute a global service delivery platform for many industries, including health care, and could offer the potential to broadly diffuse more intensive self-monitoring, feedback, and self-management tools at reduced cost. The increase of mobile health care devices and software has greatly extended self-care capabilities across the spectrum of health care activities. Current smartphones allow users to easily access a wide range of health educational materials and services anywhere at any time. Health apps running on smartphones or other portable devices enable the remote monitoring of vital parameters to diagnose health problems, track responses to treatments of chronic illnesses, detect drifts from the control condition, and provide early warning signals of potentially dangerous changes in a patient’s health status. In this context, mobile phone interventions have become increasingly popular in the global health and development sectors as a potentially inexpensive and effective way to communicate with and deliver services to people. In particular, telemedicine offers the possibility of the prolonged longitudinal monitoring of patients, which would help to highlight early and subclinical signs of diseases or complications that could manifest at a later date, and thus allow therapeutic intervention at a stage of higher reversibility. In this field, where CR is needed but insufficiently implemented, alternative rehabilitation models using new resources of communication technologies (eg, mobile- and web-based platforms, wearable sensor devices) is a new challenge to deliver supervision, education, and counseling. With these technologies, it will also be possible to consent and improve continuity of care in vulnerable populations.

HBCR, as a branch of telehealth, can be optimized and facilitated with the use of remote telemedicine devices [18]. However, some studies demonstrated that there are no substantial differences in rehabilitation programs practiced using telemedicine or according to conventional protocols in terms of functional improvement, control of risk factors, well-being, and rehospitalization and survival rates [19-21]. In contrast to CBCR, HBCR associated with telemedicine appears to be able to improve some of the shortcomings of traditional rehabilitation programs [22]. These traditional services are often associated with high costs, both to the health care system and to the patient who must make multiple trips to the rehabilitation center during the treatment process or needs to be admitted directly to intermediate care facilities dedicated to rehabilitation only.

The Box 2.0 Study Protocol is an ongoing trial with the objective of achieving the early diagnosis of complications after cardiac surgery (for atrial fibrillation, heart failure, and surgical wound infections) through the application of telemedicine and mobile devices (ie, smart technology) [23]. In particular, the Box 2.0 study is focused on the use of smartphone apps for the remote assessment of certain vital signs, including oxygen saturation, electrocardiographic tracing, arterial blood pressure, body temperature, body weight, and actigraphy. In general, the availability of remote monitoring of the patient makes it possible to customize CR programs according to the limits and functional capacity of each patient, thereby increasing adherence to the suggested rehabilitation program. In practice, telemedicine allows the realization of a patient-centered rehabilitation approach, which is an essential element of precision medicine [24]. This practice aims to encourage the independence of patients, including older adults, which would allow them to stay in their familiar environment while maintaining an acceptable quality of life.

Although HBCR based on a patient-centered perspective is capable of improving the participation rate, the effectiveness and achievement of the program depend on the patient’s attitude toward technology. In recent years, technology use in health management has involved populations of all age groups; however, the age group of 65-74 years represents the principal consumers of health digital tools, with higher adoption than found among those aged 18-34 years. Specifically, in the cardiology field, cardiac telerehabilitation is a mobile health tool that uses telecommunication technologies such as smartphone apps, wearable devices, and video consultations to propose remote CR services to increase convenience for patients while reducing health care costs. In addition, HBCR may bypass several barriers at the patient level, including transport difficulties; at the health care professional level, such as low endorsement; and at the health care system level, mainly due to limited facilities available providing this method. Furthermore, administering rehabilitation pathways outside the hospital environment by using remote monitoring and communication devices with patients would enable the acquisition of data (eg, heart rate during exercise and daily physical activity) in the context of the patient’s daily routine; thus, the data collected will be more realistic, enabling designing a more personalized treatment program.

### Study Aim and Objectives

The Innovation in Postoperative Rehabilitation Training and Monitoring (IPOTERI) study aims to create a tailored CR intervention using a mobile platform to support treatment during the postsurgery period with the goal of achieving the improvement of clinical and functional parameters and quality of life. The overall objective of the project is the analysis, design, implementation, and testing of a composite and integrated system for postsurgical rehabilitation therapies, specializing in cardiac surgery.

The project envisages the development and industrialization of a monitoring system that applies telemedicine techniques, combines microservices with innovative devices (ie, wearable and minimally invasive devices), and enables the continuous monitoring of the patient’s main vital parameters to provide real-time feedback during the execution of the rehabilitation exercises assigned to the patient in the individual phases of the rehabilitation therapy. This will be achieved through the implementation of software systems capable of not only supporting the rehabilitation pathway but also of enriching the program with innovative devices and eHealth services. This system can help to support additional and alternative pathways to traditional hospital pathways and will be further capable of integrating artificial intelligence (AI) techniques that support physicians in telemonitoring.

The hypothesis of the clinical study is that processing of data, both qualitative and quantitative, collected at home during the postsurgery period using eHealth devices, together with clinical data related to the perioperative time window using AI algorithms will be an effective approach for personalizing and then optimizing a postsurgery CR path. We here describe the protocol for the design, realization, and clinical testing of a composite and integrated system for postsurgical rehabilitation therapies at home focused on cardiac surgery.

## Methods

### Ethical Considerations

The research and ethics described in this study have been reviewed and approved by the institutional review board of the Ethics Committee of the Vast Area Northwest of Tuscany for Clinical Trials (protocol 20406). All procedures performed in the pilot study will be in accordance with the ethical standards of the institutional and/or national research committee and with the 1964 Helsinki Declaration and its later amendments or comparable ethical standards. All participants will provide written informed consent after a thorough review of procedures and questions and will be informed of their opportunity to opt out of the study at any time. All study data will be deidentified. No participation fee will be given to the patients, and all patients will enroll in the study voluntarily.

### Setting

The IPOTERI project is a public-private partnership comprising a multidisciplinary team, including research centers, health care facilities, technological enterprises, and cybersecurity experts.

### General Aspects of the IPOTERI Rehabilitation Platform

#### Platform Design Process and Overview

To define the best configuration of mobile support services for patients, we used the following methodology:

1. Formative research to determine the best way to implement HBCR, including a literature review, retrospective analysis of clinic data, and evaluation of eHealth devices potentially included in HBCR.
2. Development of the HBCR intervention: we implemented a software system that can support the rehabilitation pathway, but also enriched it with innovative devices and eHealth services that support the doctor in telemonitoring.
3. Pilot feasibility study: we will conduct a pilot study with patients who will receive the intervention to provide a context for testing the developed systems on a larger population sample.

**Figure 1:**
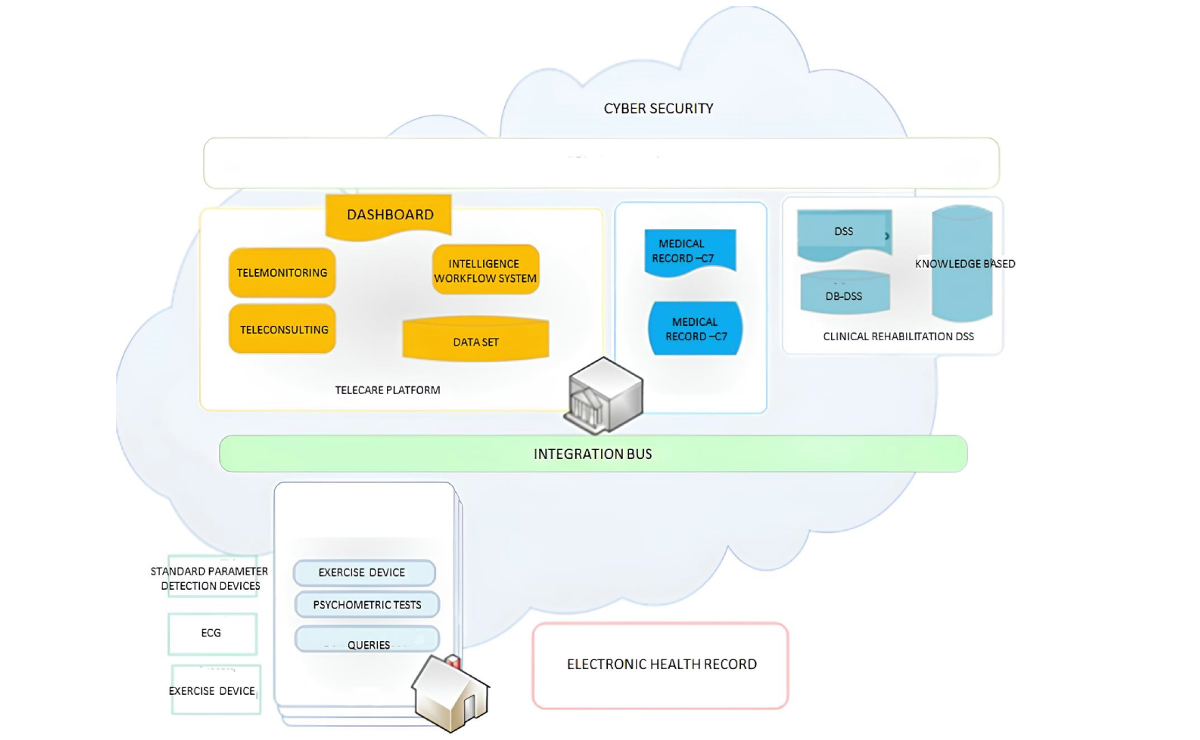
General architecture of the IPOTERI system. DSS: decision support system; C7: medical records; ECG: electrocardiogram.

The components of HBCR consist of a telemonitoring platform, an intelligent home station, and a decision support system (DSS) (Figure 1).

#### Telemonitoring Platform

The IPOTERI platform guarantees continuity of postoperative care and includes the following main functions: (1) activation of the telemonitoring process with detection of vital parameters, also in continuous mode, according to a protocol set by the doctor; (2) monitoring of rehabilitation activity, with feedback on the quality of exercise; (3) management of alerts for home events; and (4) verification of the adequacy of the patient’s vital parameters and adherence to the proposed rehabilitation program through AI techniques.

The IPOTERI platform is divided into different levels: (1) the at-home level with the Intelligent Home Data Acquisition System; (2) the territorial level with the “Telecare Platform” system, a centralized system for collecting and analyzing data from patients’ homes and hospital records; (3) the central level with the intelligent BUS system for connecting the components of the overall system; and (4) the hospital level with an integrated electronic medical record system that operates autonomously in the hospital and manages the clinical data of patients enrolled in the study.

#### Smart Home Station

**Figure 2:**
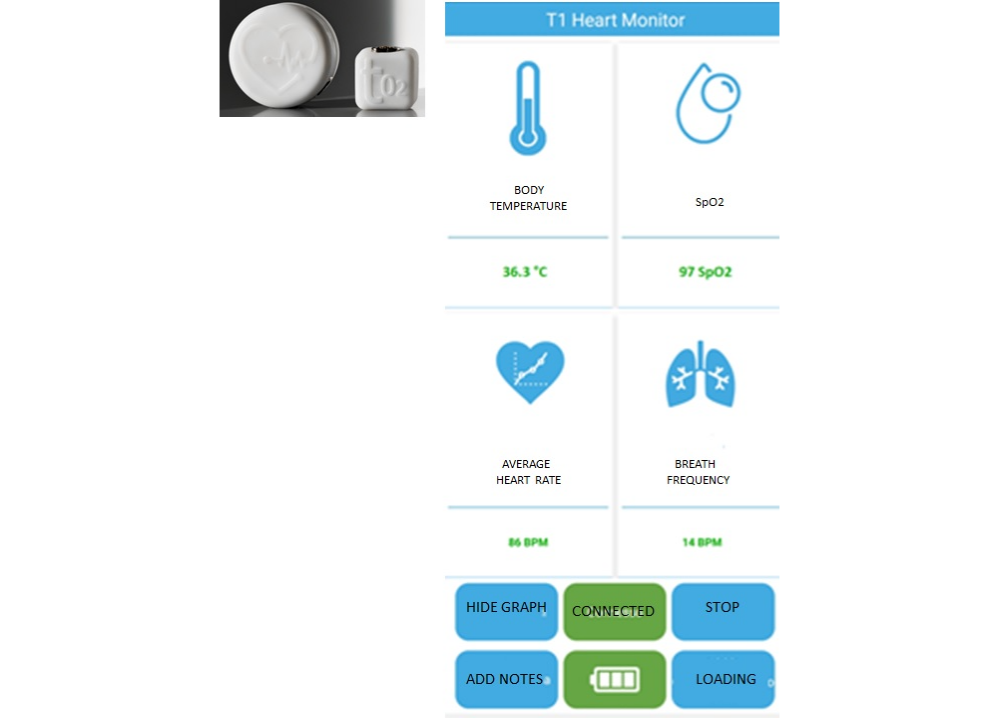
Smart home station. BPM: beats per minute; SpO2: peripheral capillary oxygen saturation.

The smart home station (SHS) is based on an Android app with a user-friendly interface for the self-recording of patients’ vital signs. The interactive part is supported by a dashboard, as shown in Figure 2. From this dashboard, the patient can access the planning of rehabilitation activities and their illustrations by means of videos and also can request a video consultation or participate in activities planned by the doctor. The SHS is also able to integrate data and information from wearable and noninvasive devices to enable the continuous monitoring of vital parameters and support the performance of assigned physical rehabilitation activities. The devices consist of a wearable, noninvasive sensor capable of continuously recording vital parameters such as electrocardiogram (ECG), heart rate, blood pressure, and oxygen saturation, and a weight balance to detect weight variations. The SHS consists of medical devices integrated with two apps: eVoDroid that is integrated with the weight balance and Umana that is integrated with the Umana T1 sensor for ECG monitoring. The eVoDroid system is an app used to receive data via Bluetooth synchronization with integrated devices; the app detects the sensor and the acquired data are displayed in the appropriate fields. It is possible to repeat the measurement, confirm it, and cancel it. There is also a manual data screen, allowing the user to add the data directly. After specifying the type of measurement, the obtained values can be input in the editable fields provided. The Umana app allows data to be received via Bluetooth synchronization with the T1 heart monitor. Once the connection is established, continuous real-time monitoring of the ECG signal and the display of other parameters, including heart rate variability, oxygen saturation, and blood pressure, are possible. The saved data are sent to a server for further analysis of the detected signal.

**Figure 3:**
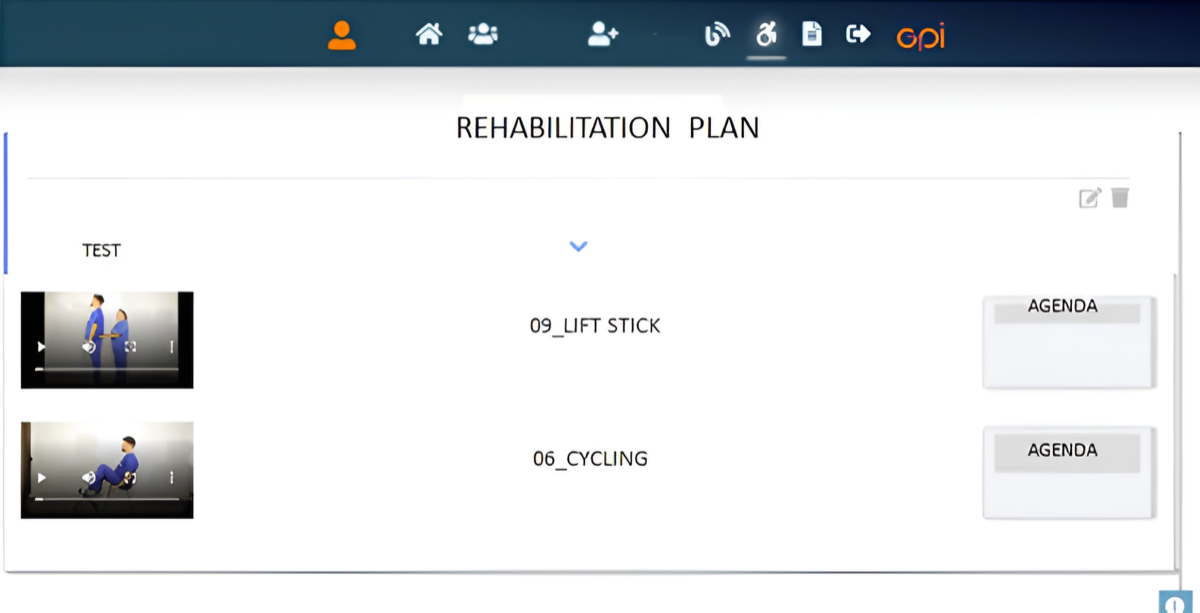
Telecare Platform for access to rehabilitation plans and related video tutorials.

At the territorial level, a dedicated “patient portal” has been defined in the Telecare Platform to carry out different activities (eg, view the agenda, carry out televisits, request a consultation) related to the rehabilitation plans along with associated video tutorials (Figure 3).

#### Clinical Rehabilitation DSS

**Figure 4:**
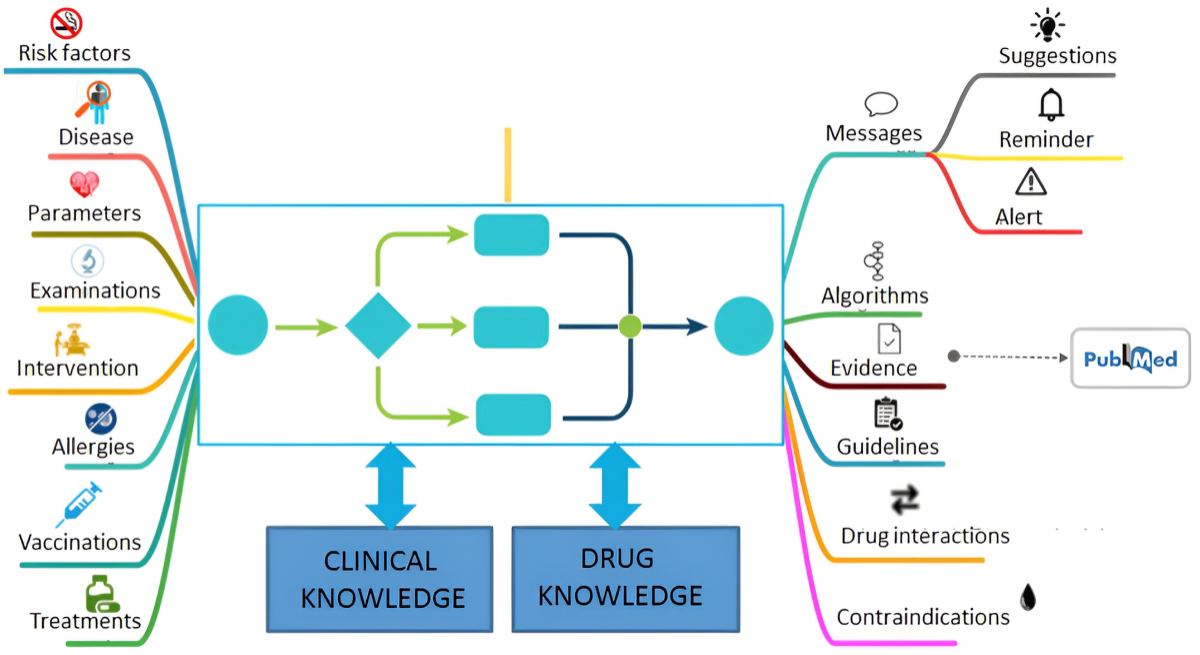
Clinical rehabilitation decision support system.

The DSS facilitates the analysis of data collected during the telemonitoring process with hospital clinical data. The DSS communicates with hospital medical records (Figure 1) to transmit alerts and specific support information for the formulation and updating of the treatment and care plan (Figure 4).

### Pilot Test

#### Study Design and Aims

In the pilot test, patients will be enrolled among those receiving coronary-artery bypass grafting without complications in the postoperative period. During this pilot study, we will receive reports of several technical issues that could affect the functionality of the system. Thus, the data obtained in the pilot study will serve as a guide in the development of a telemonitoring system, including a software and hardware platform based on telemedicine monitoring microservices, to be applied within the project to a wider population.

#### Pilot Population to Test the IPOTERI Platform

The study participants will be divided into two groups: (1) an HBCR group and (2) a control group with the same clinical profile. The HBCR group will include all patients who will undergo cardiac surgery procedures and will be submitted to telemonitoring. The control group will be established to have the same clinical profile as the HBCR group but without telemonitoring to serve as a comparison group. The inclusion criteria are as follows: 18 years and older, no major complications after surgery, and good internet connectivity at home. The main exclusion criteria are stage 4 renal failure or undergoing dialysis treatment, overt chronic respiratory insufficiency, previous cerebral ischemic or hemorrhagic event, and cognitive impairment. Allocation to the HBCR group will be based on the patient’s preference.

#### Procedure

After obtaining informed consent, preintervention clinical data will be collected, including anamnestic and instrumental data as well as predischarge data (Figure 4). At 7 to 21 days after discharge, patients will return to the hospital and will be submitted to a functional assessment by performing an aerobic exercise test, the 6-minute walking test (6MWT), according to a standard protocol. The patient will walk along a flat, hard, and measured corridor, with cones marking the turning points set by a physiotherapist [25]. The heart rate, distance traveled, arterial saturation, symptoms, and blood pressure will be monitored at the beginning and end of the exercise according to the normal clinical practice of the 6MWT. The step rate, expressed as the number of steps per minute, and the total number of steps will also be recorded. Heart rate and step rate will be recorded at the onset of symptoms and in the early stages of the exercise. The pace will be measured at the onset of symptoms and in the first 3 minutes of exercise according to the exercise table prescription.

Quality of life will be assessed based on the Psychological General Well-Being Index questionnaire [26]. During the entire monitoring period (12-14 weeks), a patient, doctor, or rehabilitator will be able to activate the teleconsultation services via a home kit. At the end of the study period, patients will undergo hematochemical examinations, echocardiogram, and functional assessment with the 6MWT. Patients who will be recruited and included in the personalized rehabilitation plan will be instructed on how to perform the functional test at home, which is aimed at assessing their rehabilitation progress and to help customize the rehabilitation program. The patient will be able to choose an exercise modality involving free walking outdoors or indoors, using an exercise bike, or a walker. If the patient performs the aerobic exercise by walking, they will be required to maintain a specific cadence or heart rate throughout the exercise. The rehabilitation program will be set based on these measurements at 60% of the walking cadence or maximum frequency reached for the first week, 70% for the second week, 80% for the third week, and 90% for the fourth week. The patient should exercise for at least 20 minutes in the first week and for at least 30 minutes per day in the following weeks. If the patient has difficulty maintaining the prescribed training goals, they will have the opportunity to interact with the doctor who can either maintain the current step increase in activity or go back one step. The rehabilitation exercises will be monitored and customized during the rehabilitation pathway through feedback with the rehabilitation therapist who will have the possibility to connect with the patient and review the execution of the exercises through the devices in the kit.

During this period, vital parameters such as ECG, respiratory rate, blood pressure, body temperature, and oxygen saturation will be recorded. All data from the rehabilitation phase will be collected and stored on the information and communications technology (ICT) platform and made available to the patient via a tablet gateway connected to the data collection center. The medical and rehabilitation staff may consult the acquired data to modify the rehabilitation program based on cardiac parameters, exercise perception, and general well-being.

At the end of the rehabilitation period, patients will undergo routine hematochemical examinations, echocardiogram, and functional assessment by the 6MWT, and they will complete the quality of life questionnaires again. In the 4th week, the patient will be tested at home by performing a walk at the maximum possible speed on a flat surface, either outdoors or indoors, for 6 minutes. During this exercise, the system will measure the continuous heart rate, respiratory rate, step rate, and oxygen saturation, and the data obtained will be used by the doctor and therapist to assess any changes in the exercises compared to the baseline examination. Based on this assessment, the doctor could decide to increase the intensity of the aerobic exercise to be performed by the patient, as described above. All data from the rehabilitation phase will be collected and stored on the ICT platform and made available to the patient via a tablet gateway connected to the data collection center. These acquired data will be available for consultation by medical and rehabilitation staff to modify the physical rehabilitation program on the basis of cardiac data, perception of effort, and general well-being.

At the end of the trial, a questionnaire will be filled out to assess the ease of acquisition of the proposed system, the ease of data transmission, and the level of comfort in managing rehabilitation with the help of the devices for the monitored patients. In addition, intervention satisfaction will be assessed with the following two items: “Overall, how satisfied are you with the IPOTERI tool?” and “How satisfied are you with the possibility of jointly participating in the IPOTERI therapy?” Fatigue severity will be assessed using the Borg rating of perceived exertion scale [27]. The scale is a very simple numerical list. Participants are asked to rate their exertion on the scale during the activity, taking into consideration feelings of physical stress and fatigue, disregarding any factor such as leg pain or breathlessness but focusing on the whole feeling of exertion.

#### AI Tools

The application of data-driven, machine learning (ML)–powered AI tools to the data collected will help to determine patterns, assess patient stratification, or compute meaningful indices for the clinicians to interpret. In the case of an insufficient population size, data *verticality* (ie, the amount of data *per patient*), or data heterogeneity, specific ML techniques will be used to enable the subsequent definition and testing of clinically relevant tasks once the data become sufficient. More precisely, an important step will be to provide appropriate *patient embedding,* which is paramount to unlock the ability to quickly and easily define and test prediction tasks over the patient population at a later date. *Embedding* a patient’s data involves converting all of the data available for a patient into a fixed-sized vector (ideally of smaller size), which can be used to train ML models (ie, classifiers and clusters) once a sufficient number of patients are available for formal analysis.

Embedding is often performed by *encoders*, which are neural networks that are trained to perform a relatively straightforward task (eg, perform the identity function in the case of *autoencoders*), featuring an architecture with a relatively small internal layer of nodes. These nodes are therefore forced by the training process to “encode” the information presented at the input stage in a fashion that is appropriate to perform the task at hand. Encoders are used in lossy compression such as for feature extraction and several other tasks. In this study, we can use encoders to obtain a compact and actionable patient representation among all heterogeneous data types available for each patient.

## Results

The pilot test started in June 2023 (protocol number 20406/2021) including 50 patients who will be monitored for 12-14 weeks using the developed platform, as described in the Procedures subsection of the Methods section.

## Discussion

### Projected Significance

This pilot study is designed to assess the acceptability, potential efficacy, and potential working mechanisms of the IPOTERI platform, a web-based tool for a postsurgery CR intervention.

To our knowledge, IPOTERI represents the first pilot experiment of the Italian experience of an HBCR program associated with telemedicine monitoring in a patient group who underwent cardiac surgery. The aim of the IPOTERI project is to develop a home-based patient telemonitoring system with the final goals of reducing the number of hospitalizations and outpatient visits, improving the communication between patients and hospital staff, and improving the psychological and emotional dimensions of CR. In the pilot study, we will provide a comprehensive description of the different components of the IPOTERI platform and the first release of the platform from patient enrollment to the rehabilitative program. The availability of remote patient monitoring will make it possible to customize CR programs according to the individual patient’s functional limitations and capabilities. In turn, this would have the advantage of increasing adherence to rehabilitation programs.

In practice, telemedicine enables the realization of a “patient-centered rehabilitation approach,” which is an essential element of precision medicine [28]. Moreover, prolonged monitoring of multiple physiological parameters of the patient can facilitate establishment of a large data bank, both in qualitative terms (number of variables) and quantitative terms with the same variable monitored over time. The accumulation of big data will necessitate the application of ML algorithms to integrate and analyze the data with a complex and dynamic approach. In this field, AI represents a potential new avenue for the integrated analysis of clinical, instrumental biohumoral data acquired longitudinally that are currently in the DSS along with data collected at single time points.

The requirement for AI in this context is becoming increasingly relevant in the age of telemedicine, which will facilitate the collection of a considerable amount of data, especially in the case of cardiovascular medicine given the complex and multifactorial physiopathological mechanisms underlying CVDs [29,30]. Scientific evidence already exists on the potential applications of AI in cardiology, using both structured and unstructured data, including data obtained from cardiac imaging methods and from eHealth devices [31-34]. For example, a recent position paper of the European Preventive Cardiology Association emphasized the usefulness of AI for the development of algorithms to improve an individual’s response to exercise so as to optimize CR in the prevention and treatment of CVDs [35].

Furthermore, the introduction of AI techniques has made it possible to develop predictive variables for outcomes of interest such as adherence to a rehabilitation program and quality of life, which would otherwise likely be neglected because these have not been considered conventional clinical parameters [36].

### Limitations

Although IPOTERI is a noninvasive technique offering a highly novel and potentially beneficial rehabilitation program, some patients may not be open to adopting this different approach and may prefer other traditional treatments. Moreover, although IPOTERI might improve patients’ perceived well-being as the patient feels monitored, the scalability of this approach to clinical parameters is still unclear. There is a potential limitation related to the approach that could affect the adherence to the rehabilitation protocol. The results, rather than being related to methodological correctness, may be related to the usability of the different devices by the patients, who are often disinclined with technology. One way to reduce this problem will be to have the protocol managed by highly qualified operators, training patients appropriately, and enrolling them voluntarily according to a certain predisposition to using such devices.

### Conclusions

The CR tool presented here could be feasible, safe, and comparable to the traditional in-hospital rehabilitation approach, indicating that rehabilitation after cardiac surgery can be carried out at home if planned with an integrated and user-friendly telemedicine service. The IPOTERI approach based on the processing of data, both qualitative and quantitative, recorded during the use of telemedicine monitoring devices at home by a patient during rehabilitation following cardiac surgery, together with clinical data referring to the perioperative and postoperative periods could have potentially positive effects on adherence to the rehabilitation program and clinical improvement, as well as in terms of quality of life. This project aims to evaluate the impact of telemedicine platforms on patients during postoperative rehabilitation. The possibility of adopting the same program in different contexts warrants future studies on a larger population to explore the actual effectiveness of telemedicine-based CR programs. The IPOTERI platform will be tested in only patients with a stable status; thus, future developments could consider the application of this platform in the management of patients with postoperative complications.

Studies are also needed to assess the possibility of adopting the same program in different clinical settings, considering the possible implementation of hybrid CR models including components of both CBCR and HBCR. 

## Abbreviations

6MWT: 6-minute walking test
AI: artificial intelligence
CBCR: center-based cardiac rehabilitation
CR: cardiac rehabilitation
CVD: cardiovascular disease
DSS: decision support system
ECG: electrocardiogram
HBCR: home-based cardiac rehabilitation
ICT: information and communications technology
IPOTERI: Innovation in Postoperative Rehabilitation Training and Monitoring
ISYDE: Italian Survey on Cardiac Rehabilitation
ML: machine learning
SHS: smart home system
